# Double intussusception secondary to Meckel’s diverticulum in a seventeen-year-old female: a case report

**DOI:** 10.11604/pamj.2020.37.175.26446

**Published:** 2020-10-22

**Authors:** Feras Sendy, Thibaut D’escrivan, Anthony Joubert, Nicolae Fetche

**Affiliations:** 1 Department of Obstetrics and Gynecology, University Hospital Center Estaing, Clermont Ferrand, France,; 2 Faculty of Medicine, University Clermont Auvergne, Clermont Ferrand, France,; 3 Department of Anesthesia, Hospital Center Vichy, Vichy, France,; 4 Department of Radiology, Hospital Center Vichy, Vichy, France,; 5 Department of Digestive Surgery, Hospital Center Vichy, Vichy, France

**Keywords:** Intussusception, Meckel’s diverticulum and laparoscopy

## Abstract

Meckel's diverticulum (MD) is the most common congenital malformation of the gastrointestinal tract. It rarely presents in adults and is usually asymptomatic. Attention to clinical history, examination and imaging studies are crucial for a successful diagnosis. A 17-year-old female presented with vomiting and acute peri-umbilical abdominal pain. Ultrasound examination showed an intussusception measuring 3.2cm in diameter and over 8cm in length. Exploratory laparoscopy showed two ileal intussusceptions. The first was reduced via laparoscopy; the second appeared suspicious for MD and ultimately required a mini-laparotomy for reduction and resection of the MD. Ultrasonography is a useful modality in the presence of perforation, occlusion, hemorrhage, neoplasia, or fistula and avoids exposure to radiation. Laparoscopic or laparoscopic-assisted mini-laparotomy is the route for the resection of MD. The choice depends on the clinical presentation and surgeon expertise. A careful history and physical examination are vital factors in diagnosis and treatment MD.

## Introduction

In 1809, Meckel´s diverticulum (MD) was described as a vestigial embryonic remnant of the omphalomesenteric duct [1]. It is more commonly identified in children, as adults are rarely symptomatic [2]. It is the most common congenital malformation of the gastrointestinal tract and represents 2-4% of the general population [3]. It is, therefore, most often discovered incidentally in adults with several potential management approaches. Herein, we report a case of double ileal intussusception that was successfully treated by laparoscopic-assisted mini-laparotomy after immediate exploratory laparoscopy.

## Patient and observation

**Figure 1:**
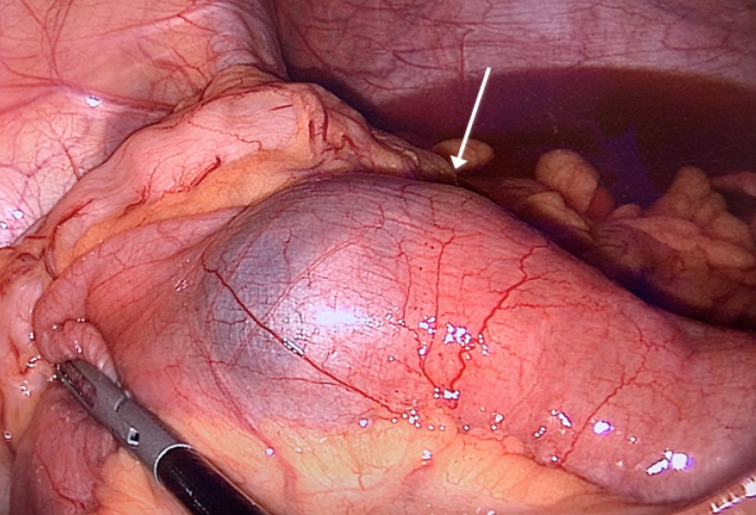
distal intussusception (arrow)

**Figure 2:**
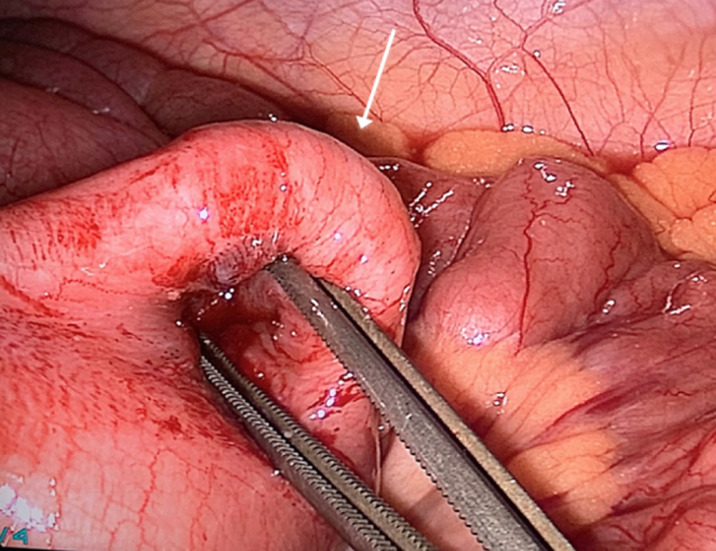
proximal intussusception with the visible base of the MD (arrow)

A 17-year-old female presented to the emergency department with vomiting and acute peri-umbilical abdominal pain for 12 hours. She had no symptoms of obstipation. Her past medical history was unremarkable. On physical examination: the patient had normal vital signs and an unremarkable abdominal examination with no tenderness. Laboratory workup showed an elevated WBC count of 20.03 10^9^/L, hemoglobin level of 15.0 g/dL, platelet count 354 10^9^/L, and a C-reactive protein of <3.0 mg/L. Ultrasound examination showed a suprapubic intussusception measuring 3.2cm in diameter and extending approximately 8cm in length with significant liquid stasis proximally. Otherwise, no intra-abdominal, pelvic, or retroperitoneal abnormalities were identified. After informed consent was obtained, we took the patient for urgent exploratory laparoscopy. We discovered a double ileal intussusception with the presence of multiple mesenteric lymph nodes without lymphadenopathy. The first was at the terminal ileum followed by a second intussusception, which was 30cm from the terminal ileum. The distal intussusception of 5cm in length was (Figure 1) easily reduced without inadvertent injury. The second intussusception of 15cm in length (Figure 2) had signs of ischemia (color change and venous congestion) with a high risk of perforation and its reduction via the laparoscopic route was difficult.

**Figure 3:**
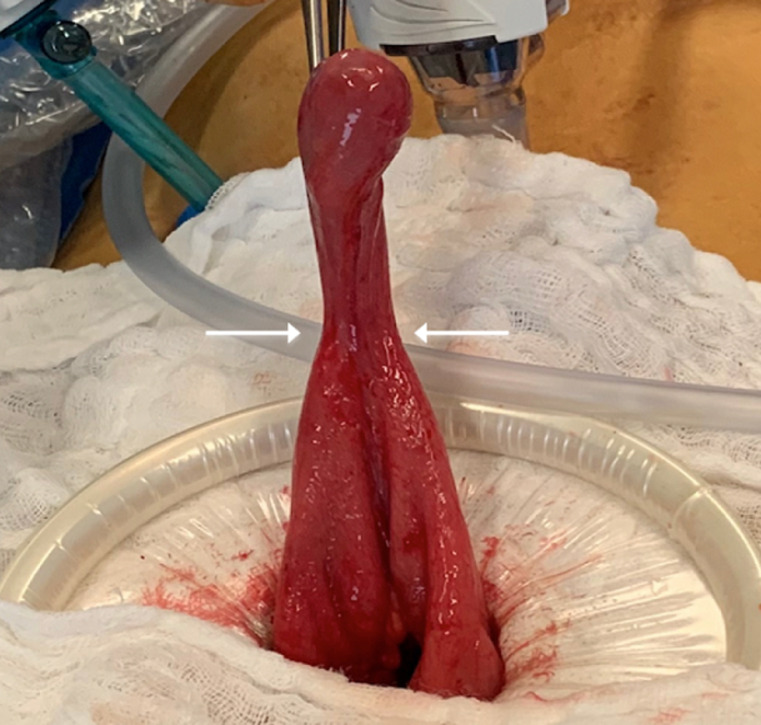
Meckel's diverticulum prior to resection at the base (arrows)

**Figure 4:**
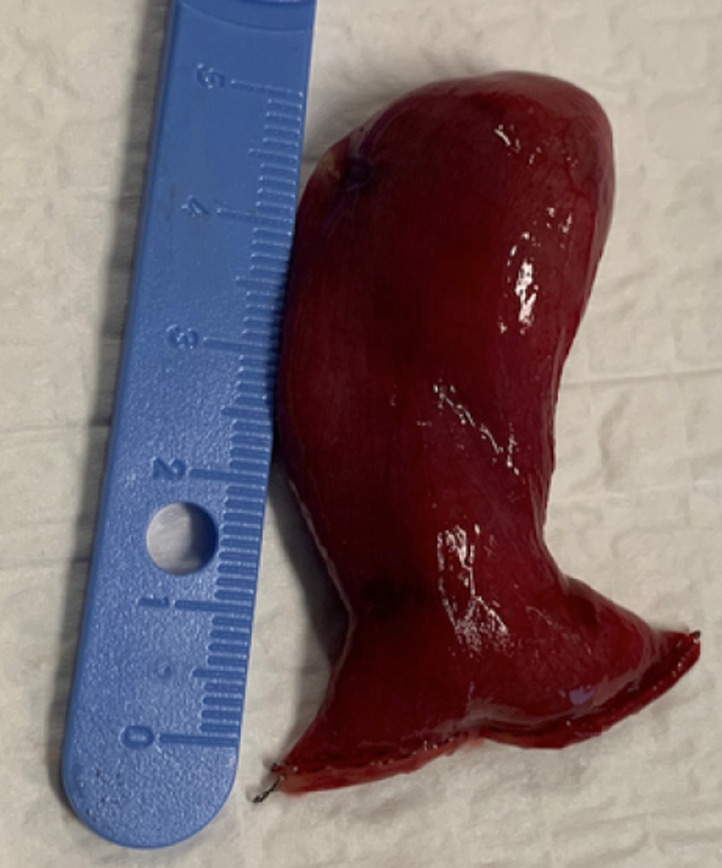
resected Meckel´s diverticulum measuring 5cm

Therefore, we decided to reduce it through a mini-laparotomy via Pfannenstiel incision. After reduction of the 5cm MD, the bowel perfusion improved with return of normal color and contractility. Resection of MD is indicated upon discovery in any circumstance, especially in the setting of an intussusception with the MD as a pathologic lead point (Figure 3 and Figure 4). The patient was discharged on the second post-operative day following an uncomplicated hospital course. Histological examination revealed the presence of gastric heterotopia with no malignancy and confirmed the diagnosis of Meckel´s diverticulum. One month later, the follow up appointment was unremarkable and the patient was cleared for unrestricted activity.

## Discussion

MD is most often asymptomatic and discovered incidentally intra-operatively, or on an imaging study. However, up to 7% of cases present with complications [3], which include perforation, obstruction, hemorrhage, neoplasia, or fistulac [4]. There is no gold standard imaging study in the absence of complications in adults. A contrast-enhanced CT scan may miss the diagnosis of MD in the absence of a complication [5]. In the presence of perforation or obstruction, a CT scan is the best imaging modality [6]. Ultrasonography has two advantages over CT scan. It is sensitive in the presence of complications and avoids radiation exposure [7].

Ectopic mucosa tends to develop in MD with the majority being gastric in up to 26% of cases [8]. Other types of ectopic tissue such as pancreatic, duodenal, colonic, endometrial and hepato-biliary can be seen within the MD [3]. Furthermore, the risk of metaplasia and neoplasia is not negligible as the mean annual incidence rate of MD cancer was 1.44 per 10 million populations between 1973 - 2006 (± standard deviation of 1.12) [9,10]. Two-thirds of these cases involve carcinoid metaplasia with adenocarcinoma, sarcoma, stromal tumors and lymphoma also described [9,10]. The removal of the risk of malignant degeneration is a known benefit to resection, which essential in our case.

In the literature, there are multiple surgical options for MD, which are segmental small bowel resection with primary anastomosis, wedge resection, or tangential stapling [9,11-13]. It is challenging to perform wedge resection or tangential stapling in cases of perforation or hemorrhagic ulceration. Linear tangential stapling [13], or wedge resection [5,11] are practiced even if there is a small risk of leaving heterotopic tissues [14]. In our case, we performed a mini-laparotomy followed by linear tangential stapling for the proximal intussusception due to the fact that; the reduction was difficult and risked traumatizing the bowel laparoscopically and benefit from palpation to ensure that all thickened tissues were removed.

Finally, various cases of MD are discovered incidentally. Therefore, is expectant non-operative management a reasonable alternative in adult patients? Some studies advocate for abstention for two main arguments: older individuals have fewer complications of MD, and higher risk of postoperative complications [15,16]. On the other hand, routine resection is advocated, as complications from MD itself are higher than postoperative complications [17] and the association of cancer potential [10]. Moreover, a retrospective study at the Mayo Clinic of 1476 patients investigated the risk factors of complications in MD and they concluded a combination of age less than 50 years old, male gender, length more than 2cm and macroscopic abnormalities increased complications up to 70% [8]. Thus, promoting prophylactic excision. It is debatable to operate cases of MD with incidental findings. Nevertheless, we believe that resection has better potential to decrease the risk of malignant degeneration and the occurrence of complications, especially in the presence of risk factors nowadays, with the advancement of imaging modalities and minimally invasive surgery.

## Conclusion

MD is rarely manifests in the adult population as these are predominantly asymptomatic. In a patient with atypical abdominal symptoms, however, imaging findings may alert general surgeons to the possibility of this diagnosis. It is evident that exploratory laparoscopy is vital in cases presenting with a complication of MD, yet the modality of choice in treatment is dependent on surgeons' experience and presenting complications.
